# Characterizing Cardiovascular Function In The Inflammatory Subphenotypes of Acute Respiratory Distress Syndrome

**DOI:** 10.1097/CCE.0000000000001483

**Published:** 2026-09-07

**Authors:** Minesh Chotalia, Muzzammil Ali, Ravi Chotalia, Dhruv Parekh, Jaimin M. Patel, Mansoor N. Bangash

**Affiliations:** 1 Department of inflammation and ageing, Birmingham Acute Care Research Group, University of Birmingham, Birmingham, UK.; 2 Department of Anaesthetics and Critical Care, Queen Elizabeth Hospital Birmingham, UK.; 3 Department of Cardiology, Lincoln County Hospital, UK.

**Keywords:** acute respiratory distress syndrome, hyperdynamic, hyperinflammatory, right ventricular dysfunction, right ventricular failure, subphenotypes

## Abstract

**OBJECTIVES:**

To compare transthoracic echocardiography (TTE) and cardiovascular parameters in patients with hyper- and hypoinflammatory acute respiratory distress syndrome (ARDS), derived using a validated, artificial intelligence clinical classifier. To compare overlap between latent class analysis-derived cardiovascular subphenotypes and clinical classifier-derived inflammatory subphenotypes of ARDS.

**DESIGN:**

Retrospective single-center cohort study.

**SETTING:**

University Hospital ICU, Birmingham, United Kingdom.

**PATIENTS:**

Intubated patients who received a transthoracic echocardiogram within 7 days of ARDS onset between April 2016 and December 2021.

**MEASUREMENTS AND MAIN RESULTS:**

A total of 691 patients were included, age 61 years (interquartile range, 50–72 yr), 65% male, 41% 90-day mortality rate. A total of 30% (*n* = 207) were classified as hyperinflammatory and had higher mortality (64% vs. 31%; *p* < 0.0001) compared with the hypoinflammatory subgroup. Right ventricular (RV) size and function did not differ between hyper- and hypoinflammatory subphenotypes. Instead, the hyperinflammatory subgroup exhibited a hyperdynamic cardiovascular profile with higher left ventricular ejection fraction and cardiac output. Latent class analysis identified four cardiovascular subphenotypes; RV-related profiles were predominantly hypoinflammatory, whereas the hyperdynamic cardiovascular subtype was enriched in hyperinflammatory patients.

**CONCLUSIONS:**

Inflammatory and cardiovascular subphenotypes represent complementary but distinct axes of ARDS pathobiology. The hyperinflammatory subphenotype aligns with a hyperdynamic circulation, whereas RV dysfunction may be mechanistically independent.

**KEY POINTS**

**Question:** Do clinical classifier-derived hyper- and hypoinflammatory ARDS subphenotypes differ in cardiovascular profile and overlap with latent class analysis-derived cardiovascular subphenotypes?

**Findings:** In 691 ARDS patients, there were no differences in right ventricular function between inflammatory ARDS subphenotypes. Instead, the hyperinflammatory phenotype was associated with a hyperdynamic cardiovascular state, marked by increased cardiac output and left ventricular ejection fraction.

**Meaning:** Inflammatory and cardiovascular subphenotypes represent distinct yet complementary dimensions of ARDS pathobiology, suggesting separate mechanistic pathways and supporting integrated phenotyping to improve prognostic precision and therapeutic targeting.

Inflammatory subphenotypes in acute respiratory distress syndrome (ARDS) have emerged as a key paradigm in understanding the biological heterogeneity of the syndrome (1). Seminal work using latent class analysis (LCA) to leverage cytokine and clinical data has consistently identified hyperinflammatory and hypoinflammatory phenotypes, which differ in clinical characteristics, outcomes, and, importantly, response to therapies (2–4); however, the physiology underpinning these subphenotypes remains incompletely characterized. Right ventricular (RV) dysfunction is highly prevalent and associated with mortality in ARDS (5, 6), and similarly, LCA of cardiovascular parameters have identified subphenotypes that differ in characteristics, outcomes, and response to therapy: RV dilation with preserved function, RV failure with dilation and systolic impairment, and hyperdynamic and “normal” circulation phenotypes (5, 6).

To the best of our knowledge, cardiovascular function has not been assessed in these inflammatory subphenotypes, and the overlap between inflammatory and cardiovascular subphenotypes is unknown. This would enhance their physiologic characterization and could provide mechanistic insights into the pathways linking hyperinflammation to mortality. It also remains unclear whether inflammatory burden drives the development of cardiovascular phenotypes in ARDS.

A study by Pensier et al demonstrated that an open-source artificial intelligence (AI) clinical classifier could accurately characterize inflammatory subphenotypes across the first 7 days of ARDS using only routinely collected clinical data (7). We applied the AI clinical classifier to a previously published ARDS cohort where every patient had received a transthoracic echocardiogram (TTE) to classify patients as hyper- or hypoinflammatory (6). We then compared cardiovascular/TTE variables between the inflammatory phenotypes and assessed overlap between inflammatory and LCA-derived cardiovascular subphenotypes.

## MATERIALS AND METHODS

This retrospective cohort study was approved by the hospital’s Institutional Review Board (RRK-7414, “Latent Class Analysis in Sepsis and ARDS,” April 28, 2021) and by the Research Ethics Committee of the NHS Health Research Authority (Integrated Research Application System [IRAS] ID 301564) and procedures were followed in accordance with the ethical standards of the responsible committee on human experimentation and with the Helsinki Declaration of 1975. All TTE’s were performed within 7 days of ARDS onset at Queen Elizabeth Hospital, Birmingham ICU, between April 2016 and December 2021 by echocardiographers with advanced accreditation only and followed the British Society of Echocardiography level 2 protocol. Clinical data used to derive phenotypes were from the day the TTE was performed. Vasopressor dose was standardized to norepinephrine equivalents (6). As the AI clinical classifier was derived and validated in intubated patients only, patients on noninvasive ventilation were excluded. Patients were also excluded if they had inadequate TTE views, severe valvular dysfunction, preexisting left ventricular (LV) or RV systolic or diastolic dysfunction, COVID-19 infection, or cardiothoracic ICU admission. LV ejection fraction was visually estimated, whereas RV: LV end-diastolic area and RV fractional area change were measured offline.

Latent class analysis approach and variable selection were kept consistent with the previously published cardiovascular subphenotype study (6) to ensure methodological consistency. The same modeling framework was reapplied within this restricted ARDS cohort that excluded nonintubated patients. For further details on latent class analysis methodology, we refer the reader to the previous publication (6).

To assess whether the association between inflammatory subphenotype and mortality differed across cardiovascular subphenotypes, a binary logistic regression model was constructed with mortality as the dependent variable, inflammatory and cardiovascular subphenotypes as independent variables. Interaction terms between inflammatory and cardiovascular subphenotypes were included to test for effect modification. Cardiovascular subphenotypes were modeled using indicator variables with the normal cardiovascular phenotype as the reference category. Statistical significance of the interaction terms was assessed using Wald tests. For each cardiovascular subphenotype, a binary logistic regression model was constructed with cardiovascular subphenotype membership as the dependent variable and inflammatory subphenotype (hyper vs. hypoinflammatory) as the independent variable, generating odds ratios with 95% CIs. *E* values were then calculated from these odds ratios via the *E* value package in R (R Foundation for Statistical Computing, Vienna, Austria). Categorical variables are presented as *n* (%) and compared using a chi-square test. Continuous variables were presented as median (interquartile range [IQR]) and compared using a Kruskal-Wallis test. A *p* value of less than 0.05 was considered statistically significant, and all tests were two-sided.

## RESULTS AND DISCUSSION

**Figure 1.**
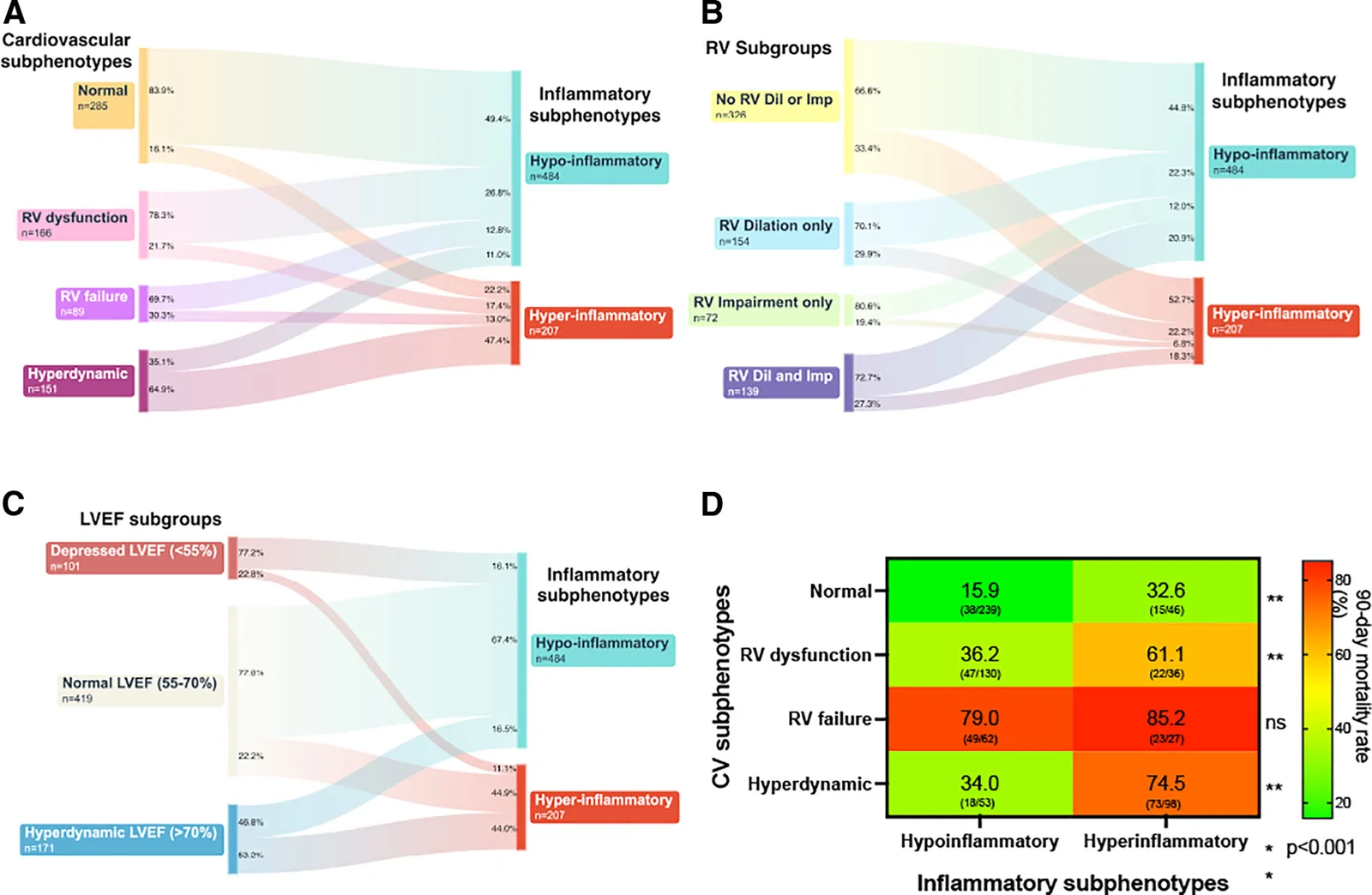
Comparing overlap and mortality between cardiovascular and inflammatory profiles. CV = cardiovascular, Dil = dilation, Imp = impairment, RV = right ventricular.

A total of 691 patients met criteria for inclusion in the study (**s-Fig. 1**, https://links.lww.com/CCX/B685), median age 61 years (IQR, 50–72 yr), 65% (*n* = 450) male, and 90-day mortality of 41.2% (*n* = 285). Most patients had moderate ARDS (49%; *n* = 338), with pneumonia being the most common risk factor for ARDS (50%; *n* = 347). Further demographics are outlined in **s-Table 1** (https://links.lww.com/CCX/B685). A total of 30% (*n* = 207) were classified into the hyperinflammatory subphenotype, which had lower platelet and bicarbonate levels and higher bilirubin, creatinine, and mortality (64.3% vs. 31.4%; *p* < 0.0001) compared with the hypoinflammatory subphenotype.

Surprisingly, TTE variables of RV size or function did not differ significantly between the hyper- and hypoinflammatory subphenotypes (**Table 1**). Instead, patients classified as hyperinflammatory demonstrated a hyperdynamic cardiovascular profile, characterized by higher left ventricular ejection fraction and cardiac index and lower systemic vascular resistance and IVC diameter and collapsibility. This persisted despite exclusion of patients with chronic liver disease (**s-Table 2**, https://links.lww.com/CCX/B685).

**TABLE 1.** Comparing Cardiovascular Parameters Between Hyper- and Hypoinflammatory Acute Respiratory Distress Syndrome Subphenotypes

Cardiovascular Parameter	All (*n* = 691), median (IQR)	Hypoinflammatory (*n* = 484), median (IQR)	Hyperinflammatory (*n* = 207), median (IQR)	*p*
RV: LV end-diastolic area	0.58 (0.51–0.70)	0.59 (0.51–0.72)	0.58 (0.50–0.69)	0.361
RV fractional area change (%)	41 (33–49)	41 (32–48)	41 (35–50)	0.300
Tricuspid annular plane systolic excursion (mm)	21 (19–24)	21 (19–24)	21 (18–24)	0.780
LV ejection fraction (%)	65 (60–70)	65 (60–65)	70 (60–75)	< 0.0001
Cardiac index (L/min/m^2^)	3.3 (2.6–4.3)	3.2 (2.5–3.9)	4.2 (3.0–5.6)	< 0.0001
Stroke volume index (mL/m^2^)	41 (32–50)	38 (31–47)	46 (34–57)	< 0.0001
Left ventricular outflow tract velocity time integral (cm^2^)	24.0 (19.7–29.4)	23.3 (19.1–27.4)	27.0 (21.0–33.1)	< 0.0001
Inferior vena cava diameter (mm)	18 (15–22)	19 (16–22)	17 (15–20)	< 0.0001
Inferior vena cava collapsibility (%)	21 (0–50)	19 (0–46)	33 (0–60)	0.0019
Vasopressor dose (µg/kg/min)	0.10 (0–0.29)	0.06 (0–0.19)	0.28 (0.12–0.48)	< 0.0001
Vasopressin	87 (12.6)	56 (11.6)	31 (15.0)	0.216
Inotrope	82 (11.9)	60 (12.4)	22 (10.6)	0.510
Central venous pressure (mm Hg)	9 (6–13)	9 (6–13)	10 (7–13)	0.445
Systemic vascular resistance index (dynes/s/cm^5^)	1528 (1029–1953)	1704 (1294–2058)	1038 (759–1587)	< 0.0001
Ratio of early diastolic transmitral flow velocity (E), to late diastolic transmitral flow velocity (A) (*n* = 562)	1 (0.9–1.3)	1 (0.9–1.3)	1 (0.8–1.2)	0.548
Early diastolic mitral annular tissue velocity lateral (*n* = 562)	10 (9–12)	10 (9–12)	11 (10–13)	0.027
Ratio of early diastolic transmitral flow velocity to early diastolic mitral annular tissue velocity lateral (*n* = 562)	9 (7–10)	9 (7–10)	8 (7–10)	0.138

IQR = interquartile range, RV = right ventricular, LV = left ventricular.

After exclusion of TTE/haemodynamic parameters due to missing values and collinearity, the same nine variables were included in latent class analysis: RV:LV end-diastolic area, LV end-diastolic area index, RV fractional area change, tricuspid annular plane systolic excursion, inferior vena cava diameter, cardiac index, central venous pressure, heart rate, and vasopressor dose. Four cardiovascular subphenotypes were identified, that resembled the ones previously described (6): class 1 (41%; mostly normal LV and RV function), class 2 (24%; mostly dilated RV with preserved systolic function), class 3 (13%; mostly RV dilation and systolic impairment), and class 4 (22%; mostly hyperdynamic cardiovascular function; **s-Tables 3** and **4**, https://links.lww.com/CCX/B685). When inflammatory subphenotypes were compared with these cardiovascular ones, RV-related cardiovascular subtypes were predominantly hypoinflammatory, whereas the hyperdynamic cardiovascular subtype was largely enriched within the hyperinflammatory subgroup (**Fig. 1*A***). This pattern was similarly observed when RV and hyperdynamic cardiovascular profiles were diagnosed based on clinical definitions (**Fig. 1**, ***B*** and ***C***).

The absence of differences in RV size and function between inflammatory phenotypes is noteworthy, given the poor prognosis RV dysfunction portends. These findings suggest that inflammatory burden alone may not be the principal determinant of RV dysfunction, which instead may represent a distinct pathophysiological pathway in ARDS, more influenced by cardiopulmonary mechanics and factors affecting pulmonary vascular resistance, including hypoxic pulmonary vasoconstriction, hypercapnia, acidosis, micro- or macro-thrombosis, and mechanical ventilation strategies. This may explain why RV dysfunction is inconsistently associated with inflammatory markers in prior studies (6). This lack of overlap reinforces the multidimensional pathophysiology of ARDS and strengthens the rationale for integrated phenotyping approaches incorporating immune, pulmonary, and cardiovascular domains to better inform prognosis and guide therapy.

In contrast, the hyperinflammatory phenotype being closely associated with a hyperdynamic circulatory state is biologically plausible and consistent with the known haemodynamic consequences of systemic inflammation, which include cytokine-mediated vasodilation and compensatory increases in cardiac output, especially evident in sepsis (8), a syndrome with notable overlap with ARDS. These findings support the concept that inflammatory burden and systemic haemodynamics capture distinct but intersecting axes of the syndrome.

Although mortality was higher in most of the cardiovascular subphenotypes when they were classified as hyperinflammatory compared with hypoinflammatory (**Fig. 1*D***), interaction testing demonstrated a significant interaction between hyperdynamic cardiovascular subphenotype and inflammatory phenotype for 90-day mortality (*p* = 0.013), but no interaction between RV-related subphenotypes and inflammatory subphenotypes (*p* > 0.05). This suggests that inflammation has a substantial additive effect on outcome when cardiovascular function is hyperdynamic; however, in the setting of RV dysfunction, this cardiovascular collapse may directly drive mortality independent of systemic inflammation.

To assess the robustness of these findings to potential misclassification by the AI clinical classifier, *E* values were calculated. The association between hyperinflammatory subphenotype and hyperdynamic cardiovascular subphenotype was an odds ratio (OR) of 7.3; 95% CI, 5.0–10.9, and the *E* value for the point estimate was 4.9 and for the lower CI was 3.9, indicating that misclassification would need to produce greater than 3.9-fold systematic bias to nullify this finding. This is an implausible degree of error for a classifier with an area under the receiver operating characteristic curve of 0.87 (7, 9). The hyperinflammatory phenotype was not associated with RV dysfunction (OR, 1.4; 95% CI, 0.6–1.6) or RV failure (OR, 1.0; 95% CI, 0.6–1.6), with *E* values of 1.0 for both point estimates, which is consistent with the conclusion that RV-related cardiovascular phenotypes are not meaningfully driven by inflammatory burden.

This study has several limitations. Its single-center nature limits generalisability, and retrospective design introduces selection bias. TTE timing varied across the first 7 days; however, findings were preserved in TTE’s performed early (< 3 d) and late (> 3 d) (**s-Table 5**, https://links.lww.com/CCX/B685). Phenotypic classification was performed at a single time point only, providing only a snapshot of what could be a dynamic disease process, and temporal variability may have reduced associations between subphenotypes. The AI clinical classifier misclassified 22% of patients compared with parsimonious biomarker models (9); however, *E* value analysis suggests that these findings are robust to this degree of misclassification. Finally, the use of newer markers of RV function (e.g., 3D RV ejection fraction or RV global longitudinal strain) could provide a more sensitive assessment of RV dysfunction. A prospective study with serial, paired TTE and biomarker measurements is needed to validate these findings.

Altogether, these findings demonstrate that inflammatory and cardiovascular subphenotypes capture complementary but nonredundant axes of ARDS pathobiology. The concordance between hyperinflammation and hyperdynamic circulation suggests inflammation can drive cardiovascular responses, while the lack of overlap with RV dysfunction indicates there may be a separate, cardiopulmonary-mediated risk pathway.

## Supplementary Material
