# Supplementary material for: Characterizing Cardiovascular Function In The Inflammatory Subphenotypes of Acute Respiratory Distress Syndrome
Source: Crit Care Explor. 2026 Sep 7;8(9):e1483. doi: 10.1097/CCE.0000000000001483 (PMC13549566; doi:10.1097/CCE.0000000000001483)
Supplement: Supplementary file 1 [file cc9-8-e1483-s001.pdf]

S-figure 1: Flowchart for inclusion of patients in the study

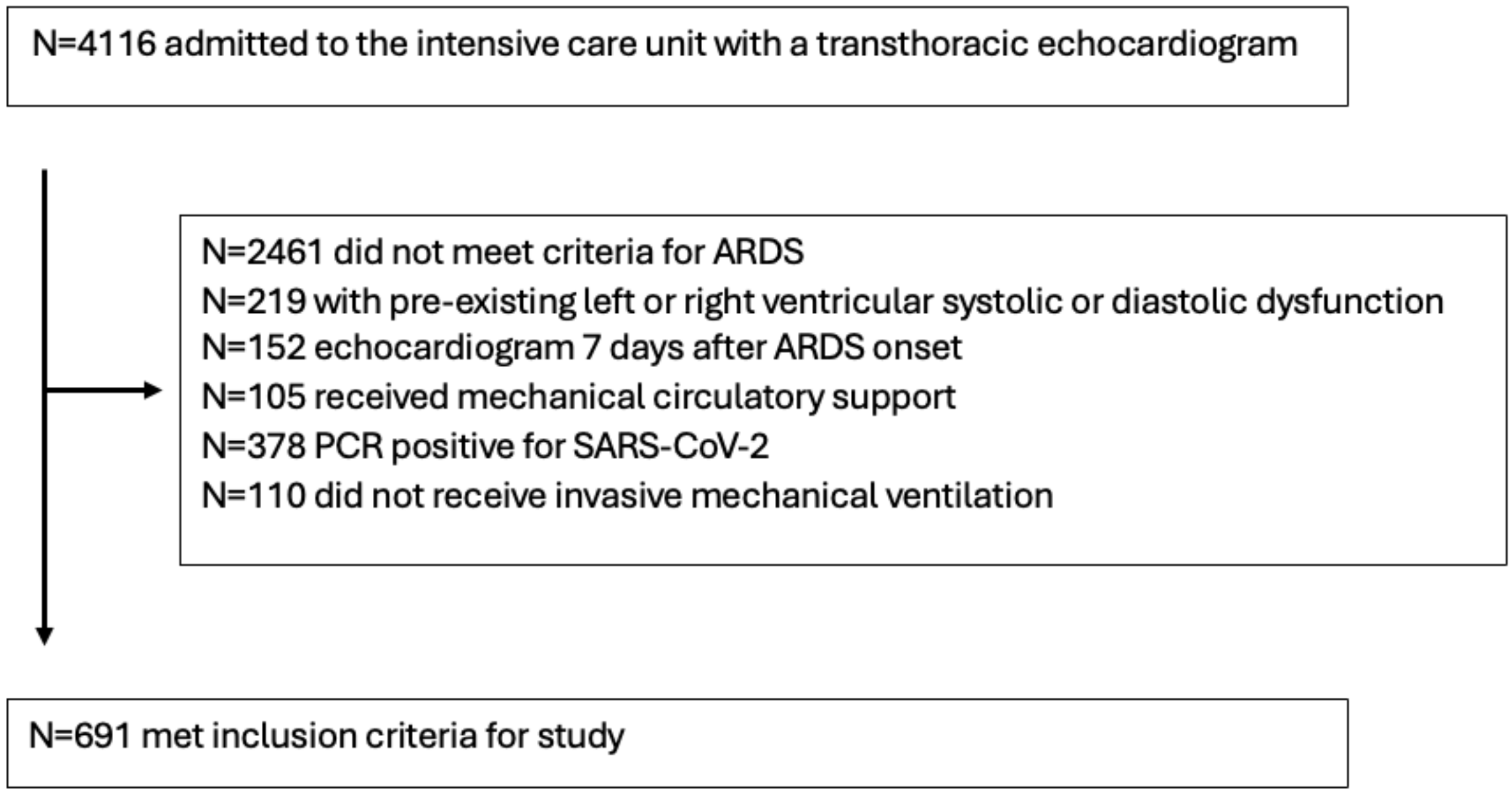

S-Table 1: Comparing additional demographics, clinical classifier and cardiovascular variables between inflammatory subphenotypes

**Parameter**	**All (n=691)**	**Hypo-inflammatory (n=484)**	**Hyper-inflammatory (n=207)**	**P Value**
**Demographics**				
Age; years	61 (50 – 72)	63 (52 – 73)	58 (42 – 69)	0.0014
Sex; % male	450 (65.1)	322 (69.4)	128 (61.8)	0.236
Day of TTE	3 (2 – 5)	3 (2 – 5)	3 (2 – 5)	0.104
ARDS severity				<0.0001
Severe	19 (2.8)	8 (1.7)	11 (5.3)	
Moderate	338 (48.9)	216 (44.6)	122 (58.9)	
Mild	334 (48.3)	260 (53.7)	74 (35.8)	
SOFA score	8 (5 – 11)	6 (4 – 8)	10 (8 – 12)	<0.0001
Risk factor for ARDS				<0.0001
Pneumonia	347 (50.2)	261 (53.9)	86 (41.6)	
Non-respiratory sepsis	182 (26.3)	90 (18.6)	92 (44.4)	
Trauma	95 (13.8)	82 (16.9)	13 (6.3)	
Aspiration	51 (7.4)	42 (8.7)	9 (4.4)	
Other	16 (2.3)	9 (1.9)	7 (3.4)	
Sepsis n (%)	529 (76.6)	331 (68.4)	191 (92.2)	<0.0001
Septic shock n (%)	155 (22.4)	83 (17.2)	72 (34.8)	<0.0001
Charlson comorbidity index	3 (1 – 4)	2 (1 – 4)	3 (2 – 5)	<0.0001

Performance status	1 (1 – 1)	1 (1 – 1)	1 (1 – 1)	0.605
Comorbidities				<0.0001
Atrial fibrillation	28 (4.1)	19 (3.9)	9 (4.4)	
Obese	141 (20.4)	94 (19.4)	47 (22.7)	
Cancer	121 (17.5)	77 (15.9)	44 (21.3)	
Ischemic heart disease	94 (13.6)	70 (14.5)	24 (11.6)	
Peripheral vascular disease	35 (5.1)	26 (5.4)	9 (4.4)	
Hypertension	162 (23.4)	112 (23.1)	50 (24.2)	
COPD / Asthma	111 (16.1)	91 (18.8)	20 (9.7)	
TIA/stroke	59 (8.5)	46 (9.5)	13 (6.3)	
Chronic kidney disease	77 (11.1)	42 (8.7)	35 (16.9)	
Diabetes mellitus	97 (14.0)	64 (13.2)	33 (15.9)	
Chronic liver disease	91 (13.2)	46 (9.5)	45 (21.7)	
None	167 (24.2)	132 (27.3)	35 (16.9)	
**AI Clinical classifier**				
Bilirubin (umol.L^{-1})	14 (8 – 31)	10 (6 – 16)	49 (17 – 152)	<0.0001
Creatinine (umol.L^{-1})	87 (57 – 144)	74 (53 – 106)	150 (89 – 216)	<0.0001
Albumin (g.L^{-1})	27 (23 – 31)	27 (23 – 31)	27 (23 – 32)	0.414
Glucose (mmol.L^{-1})	9.1 (6.0 – 11.8)	8.9 (6.0 – 11.7)	9.1 (6.0 – 12.3)	0.207
Sodium (mmol.L^{-1})	140 (136 – 144)	141 (137 – 145)	138 (135 – 141)	<0.0001
Haematocrit (%)	41 (32 – 51)	41 (33 – 51)	40 (32 – 49)	0.849
White blood cell count ($x10^9.L^{-1}$)	13.0 (9.4 – 18.4)	12.7 (9.4 – 17.8)	13.7 (9.3 – 20.1)	0.274
Platelet ($x10^9.L^{-1}$)	179 (102 – 155)	210 (144 – 302)	98 (50 – 182)	<0.0001
PaCO2; kPa	6.1 (5.3 – 7.0)	6.1 (5.4 – 7.2)	5.9 (5.1 – 6.7)	0.370
PaO2:FiO2 ratio	27 (21 – 33)	29 (22 – 34)	25 (20 – 32)	0.0018
Temperature (C)	36.7 (36.1 – 37.3)	36.8 (36.3 – 37.3)	36.5 (35.9 – 37.2)	0.0033
Respiratory rate (breath/min)	20 (17 – 22)	20 (17 – 21)	20 (17 – 22)	0.377
Heart rate (bpm)	87 (76 – 101)	85 (74 – 99)	93 (82 – 107)	0.0036
Systolic blood pressure (mmHg)	117 (101 – 139)	121 (101 – 143)	114 (97 – 133)	0.083
Urine output (ml/day)	1250 (570 – 2056)	1487 (859 – 2200)	660 (244 – 1492)	<0.0001

Minute ventilation (L/min)	9.3 (8.1 – 10.7)	9.3 (8.1 – 10.6)	9.5 (8.1 – 11.0)	0.670
Bicarbonate (mmol.L^{-1})	26 (22 – 29)	27 (24 – 30)	23 (20 – 25)	<0.0001
**Other clinical**				
Peak airway pressure; cmH2O	22 (20 – 26)	22 (20 – 27)	23 (20 – 26)	0.805
PEEP; cmH2O	7 (5 – 8)	7 (5 – 8)	6 (5 – 8)	0.968
CXR opacification	6 (3 – 8)	6 (3 – 8)	6 (4 – 8)	0.073
C-reactive protein	107 (47 – 212)	108 (52 – 212)	106 (41 – 214)	0.790
Lactate; mmol.L^{-1}	1.5 (1.1 – 2.3)	1.3 (1.0 – 1.8)	2.1 (1.5 – 3.5)	<0.0001
FiO2 (%)	45 (35 – 57)	44 (35 – 55)	50 (45 – 61)	<0.0001
Cumulative fluid balance	-776 (-85 – -1560)	-1068 (-454 – -1711)	-145 (+206 – -776)	<0.0001
pH	7.37 (7.31 – 7.42)	7.38 (7.32 – 7.43)	7.33 (7.27 – 7.39)	<0.0001
**Management**				
Prone ventilation	39 (5.6)	20 (4.1)	19 (9.2)	0.0085
Neuromucular blockade	188 (27.2)	112 (22.9)	76 (36.9)	<0.0001
Renal replacement therapy	177 (25.6)	74 (15.3)	103 (49.8)	<0.0001
90-day mortality	285	152 (31.4)	133 (64.3)	<0.0001

S-table 2: Comparing cardiovascular variables between inflammatory subphenotypes in patients without chronic liver disease

**Parameter**	**All (n=600)**	**Hypo-inflammatory (n=442)**	**Hyper-inflammatory (n=158)**	**P Value**
RV:LV end-diastolic area	0.58 (0.50 – 0.70)	0.59 (0.51 – 0.72)	0.57 (0.50 – 0.67)	0.203
RV fractional area change; %	40 (32 – 49)	40 (32 – 48)	42 (35 – 50)	0.149
Tricuspid annular plane systolic excursion; mm	21 (18 – 24)	21 (19 – 24)	21 (17 – 24)	0.357
LV ejection fraction (%)	65 (60 – 70)	65 (59 – 65)	70 (65 – 75)	<0.0001
Cardiac index (L.min^{-1}.m^{-2})	3.2 (2.5 – 4.1)	3.1 (2.5 – 3.8)	4.1 (2.9 – 5.3)	<0.0001

Stroke volume index (ml.m^{-2})	39 (31 – 48)	38 (31 – 46)	45 (33 – 55)	<0.0001
Left ventricular outflow tract velocity time integral; cm^2	24 (19 – 28)	23 (19 – 27)	25 (20 – 31)	<0.0001
Inferior vena cava diameter; mm	18 (15 – 22)	19 (16 – 22)	17 (14 – 20)	<0.0001
Inferior vena cava collapsibility (%)	20 (0 – 48)	17 (0 – 44)	33 (0 – 60)	0.0066
Vasopressor dose (mcg.kg^{-1}.min^{-1})	0.09 (0 – 0.27)	0.06 (0 – 0.18)	0.28 (0.11 – 0.56)	<0.0001
Central venous pressure; mmHg	9 (7 – 12)	9 (7 – 12)	10 (7 – 13)	0.148
Systemic vascular resistance index; dynes.sec^{-1}.cm^{-5}	1597 (1112 – 1996)	1715 (1295 – 2096)	1212 (821 – 1697)	<0.0001
E/A	1 (0.9 – 1.3)	1 (0.9 – 1.3)	1 (0.8 – 1.2)	0.114
e' lateral	10 (9 – 12)	10 (9 – 12)	10 (9 – 13)	0.501
E/e' lateral	9 (7 – 10)	9 (7 – 10)	9 (7 – 10)	0.659

s-table 3: Fit statistics following latent class analysis of cardiovascular variables

	**LL**	**BIC(LL)**	**AIC(LL)**	**Max. BVR**	**VLMR p-value**	**Class.Err.**	**Entropy R^2**
1-Cluster	-8376.0372	16869.7610	16788.0745	108.1854		0.0000	1.0000
2-Cluster	-8023.1707	16281.7143	16118.3413	51.7747	0.0000	0.0401	0.7791
3-Cluster	-7842.2276	16037.5147	15792.4552	18.1320	0.0000	0.1149	0.7219
4-Cluster	-7736.8385	15944.4230	15617.6769	12.4590	0.0000	0.1381	0.7233
5-Cluster	-7701.8121	15992.0569	15583.6243	7.0958	0.0015	0.1748	0.7085

S-table 3 legend Legend: LL = likelihood ratio. BIC = Bayesian information criteria. AIC = akaike information criteria. MaxBVR = maximum bivariate residual. VLMR = Vuoung-Lo-Mendell-Rubin test p value

S-table 4: Comparing clinical and haemodynamic variables between latent class analysis derived cardiovascular subphenotypes

Parameter	All (n=691)	Normal (n=285)	RV dysfunction (n=166)	RV failure (n=89)	Hyperdynamic (n=151)	P Value
**Demographics**						
Age; years	61 (50 – 72)	64 (52 – 73)	59 (47 – 71)	63 (53 – 76)	59 (46 – 71)	0.025
Sex; % male	450 (65.1)	188 (65.9)	110 (66.3)	62 (70.0)	90 (59.6)	0.389
Day of TTE	3 (2 – 5)	3 (2 – 5)	3 (2 – 5)	3 (2 – 4)	3 (2 – 5)	0.592
**AI clinical classifier variables**						
Bilirubin (umol.L^{-1})	14 (8 – 31)	12 (7 – 25)	14 (17 – 35)	17 (9 – 29)	16 (10 – 60)	0.0010
Creatinine (umol.L^{-1})	87 (57 – 144)	81 (55 – 128)	86 (56 – 151)	105 (70 – 175)	86 (61 – 141)	0.015
Albumin (g.L^{-1})	27 (23 – 31)	27 (24 – 31)	28 (24 – 31)	26 (22 – 30)	27 (23 – 31)	0.533
Glucose (mmol.L^{-1})	9.1 (6.0 – 11.8)	9.0 (6.0 – 11.9)	9.2 (6.1 – 12.0)	9.2 (6.1 – 11.9)	8.9 (5.9 – 11.7)	0.240
Sodium (mmol.L^{-1})	140 (136 – 144)	140 (137 – 143)	142 (137 – 146)	139 (136 – 143)	139 (136 – 143)	0.0012
Haematocrit (%)	41 (32 – 51)	40 (31 – 50)	41 (33 – 52)	41 (32 – 50)	40 (30 – 50)	0.403
White blood cell count (x10^{9}.L^{-1})	13.0 (9.4 – 18.4)	13.0 (9.4 – 18.4)	11.7 (8.8 – 16.8)	13.6 (9.8 – 19.0)	14.6 (9.7 – 20.1)	0.0137

Platelet ($x10^9.L^{-1}$)	179 (102 – 155)	205 (124 – 288)	168 (85 – 257)	177 (104 – 284)	161 (77 – 262)	0.0023
PaCO2; kPa	6.1 (5.3 – 7.0)	5.8 (5.2 – 6.6)	6.2 (5.0 – 7.5)	6.3 (5.7 – 7.9)	6.0 (5.4 – 6.7)	0.0316
PaO2:FiO2 ratio	27 (21 – 33)	29 (22 – 34)	27 (22 – 33)	25 (19 – 31)	26 (20 – 32)	0.0210
Temperature (C)	36.7 (36.1 – 37.3)	36.8 (36.2 – 37.3)	36.7 (36.3 – 37.2)	36.4 (35.9 – 37.0)	36.9 (36.1 – 37.5)	0.0054
Respiratory rate (breath/min)	20 (17 – 22)	19 (17 – 22)	20 (17 – 22)	21 (18 – 24)	20 (17 – 22)	0.449
Heart rate (bpm)	87 (76 – 101)	80 (69 – 90)	83 (74 – 95)	88 (73 – 106)	100 (90 – 111)	<0.0001
Systolic blood pressure (mmHg)	117 (101 – 139)	121 (104 – 143)	118 (102 – 140)	112 (99 – 134)	109 (94 – 128)	<0.0001
Urine output (ml/day)	1250 (570 – 2056)	1421 (773 – 2182)	1499 (701 – 2056)	778 (265 – 1470)	1015 (377 – 2060)	<0.0001
Minute ventilation (L/min)	9.3 (8.1 – 10.7)	9.2 (8.0 – 10.6)	9.2 (8.0 – 10.7)	9.3 (8.1 – 10.7)	9.4 (8.2 – 10.8)	0.0341
Bicarbonate ($mmol.L^{-1}$)	26 (22 – 29)	26 (22 – 29)	27 (23 – 30)	24 (20 – 28)	28 (24 – 30)	<0.0001
**Cardiovascular**						
RV:LV end-diastolic area	0.58 (0.51 – 0.70)	0.52 (0.46 – 0.58)	0.73 (0.65 – 0.81)	0.93 (0.77 – 1.08)	0.55 (0.48 – 0.59)	<0.0001
RV fractional area change; %	41 (33 – 49)	42 (36 – 49)	41 (34 – 48)	22 (17 – 28)	46 (39 – 53)	<0.0001
Tricuspid annular plane systolic excursion; mm	21 (19 – 24)	21 (19 - 23	22 (19 – 26)	16 (12 – 20)	22 (20 – 25)	<0.0001
LV ejection fraction (%)	65 (60 – 70)					

Cardiac index ($L.min^{-1}.m^{-2}$)	3.3 (2.6 – 4.3)	2.9 (2.5 – 3.5)	3.8 (3.2 – 4.4)	2.0 (1.6 – 2.5)	5.2 (4.3 – 6.3)	<0.0001
Stroke volume index ($ml.m^{-2}$)	41 (32 – 50)	38 (32 – 44)	45 (36 – 52)	26 (20 – 31)	52 (43 – 63)	<0.0001
Left ventricular outflow tract velocity time integral; cm^2	24.0 (19.7 – 29.4)	23 (19 – 26)	26 (21 – 30)	18 (13 – 23)	30 (25 – 36)	<0.0001
Inferior vena cava diameter; mm	18 (15 – 22)	18 (16 – 21)	20 (15 – 22)	22 (20 – 25)	15 (13 – 18)	<0.0001
Inferior vena cava collapsibility (%)	21 (0 – 50)	25 (0 – 47)	15 (0 – 48)	0 (0 – 9)	47 (5 – 74)	<0.0001
Vasopressor dose ($mcg.kg^{-1}.min^{-1}$)	0.10 (0 – 0.29)	0.07 (0 – 0.20)	0.07 (0 – 0.21)	0.20 (0.10 – 0.47)	0.25 (0 – 0.56)	<0.0001
Central venous pressure; mmHg	9 (6 – 13)	9 (6 – 11)	10 (8 – 12)	11 (7 – 15)	9 (6 – 13)	<0.0001
Systemic vascular resistance index; $dynes.sec^{-1}.cm^{-5}$	1528 (1029 – 1953)	1839 (1511 – 2240)	1321 (1030 – 1812)	853 (667 – 1041)	1948 (1556 - 2332)	<0.0001
E/A (n=562)	1 (0.9 – 1.3)	1.1 (0.9 – 1.3)	1.1 (0.9 – 1.4)	0.9 (0.8 – 1.2)	1.0 (0.8 – 1.1)	<0.0001
e' lateral (n=562)	10 (9 – 12)	10 (9 – 13)	11 (9 – 13)	10 (9 – 12)	10 (8 – 12)	0.078
E/e' lateral (n=562)	9 (7 – 10)	9 (7 – 11)	9 (6 – 10)	8 (7 – 10)	9 (7 – 10)	0.543
**Outcome**						
90-day mortality	285 (41.2)	53 (18.6)	69 (41.6)	72 (80.9)	91 (60.3)	<0.0001

S-table 5: Comparing subphenotype prevalence and cardiovascular variables in patients with early and late transthoracic echocardiograms

Parameter	All (n=691)	Early TTE (n=323)	Late TTE (n=368)	P Value

**Subphenotype prevalence**				0.303
Hypo-inflammatory	484 (70.0)	221 (68.4)	265 (72.0)	
Hyper-inflammatory	207 (30.0)	102 (31.6)	103 (28.0)	
**Cardiovascular**				0.987
Normal	285 (41.2)	134 (41.5)	151 (41.0)	
RV dysfunction	166 (24.0)	76 (23.5)	90 (24.5)	
RV failure	89 (12.9)	41 (12.7)	48 (13.0)	
Hyperdynamic	151 (21.9)	72 (22.3)	79 (21.5)	
**Cardiovascular**				
RV:LV end-diastolic area	0.58 (0.51 – 0.70)	0.58 (0.51 – 0.72)	0.58 (0.50 – 0.69)	0.543
RV fractional area change; %	41 (33 – 49)	41 (33 – 50)	40 (32 – 49)	0.212
Tricuspid annular plane systolic excursion; mm	21 (19 – 24)	21 (19 – 24)	21 (18 – 24)	0.571
LV ejection fraction (%)	65 (60 – 70)	65 (60 – 70)	65 (60 – 70)	0.118
Cardiac index ($L.min^{-1}.m^{-2}$)	3.3 (2.6 – 4.3)	3.4 (2.8 – 4.4)	3.3 (2.5 – 4.2)	0.048
Stroke volume index ($ml.m^{-2}$)	41 (32 – 50)	42 (33 – 51)	39 (31 – 48)	0.027
Left ventricular outflow tract velocity time integral; cm^2	24.0 (19.7 – 29.4)	24.3 (20.0 – 29.9)	23.7 (19.1 – 28.9)	0.058
Inferior vena cava diameter; mm	18 (15 – 22)	18 (15 – 22)	18 (15 – 22)	0.848
Inferior vena cava collapsibility (%)	21 (0 – 50)	24 (0 50)	20 (0 – 50)	0.240
Vasopressor dose ($mcg.kg^{-1}.min^{-1}$)	0.10 (0 – 0.29)	0.11 (0 – 0.30)	0.10 (0 – 0.29)	0.756
Central venous pressure; mmHg	9 (6 – 13)	10 (7 – 12)	10 (7 – 13)	0.583
Systemic vascular resistance index; $dynes.sec^{-1}.cm^{-5}$	1528 (1029 – 1953)	1530 (995 – 1923)	1511 (1092 – 2019)	0.382
E/A (n=562)	1 (0.9 – 1.3)	1 (0.8 – 1.3)	1 (0.9 – 1.3)	0.788
e’ lateral (n=562)	10 (9 – 12)	10 (9 – 13)	10 (9 – 12)	0.391
E/e’ lateral (n=562)	9 (7 – 10)	8 (7 – 10)	9 (7 – 11)	0.361
**Outcome**				
90-day mortality	285 (41.2)	135 (41.8)	150 (40.8)	0.783
